# Determination of residual DNA in decellularised aortas– towards fluorescence-based quantification of DNA purified by various methods

**DOI:** 10.1007/s11033-025-10755-1

**Published:** 2025-07-08

**Authors:** Stefan Pentzold, Mengjin Li, Ana Lucia Paz Hernandez, Uta Dahmen

**Affiliations:** Department of General, Visceral and Vascular Surgery, Research Group Experimental Transplantation Surgery, Jena University Hospital, Friedrich Schiller University Jena, Drackendorfer Straße 1, D-07747 Jena, Germany

**Keywords:** Decellularisation, DNase I, Detergent, DNA purification, DNA quantification

## Abstract

**Background:**

Decellularisation of animal tissues is a promising strategy to obtain scaffolds for tissue engineering. However, double-stranded (ds)DNA of animal origin that may reside in tissue even after harsh decellularisation causes adverse reactions in patients. Thus, precise determination of residual dsDNA is essential, but challenging since the methods used for purification may affect quantification.

**Methods:**

To elucidate the best method for purifying and quantifying residual dsDNA in decellularised vessels, rat thoracic aortas were perfused with detergents (sodium dodecyl sulfate/sodium deoxycholate) with or without DNase I. Native aortas were used as control. Three different methods for purifying DNA (tissue lysate, salting out, silica-based solid-phase) were applied to assess dsDNA by fluorescence-based methods (PicoGreen, real-time QPCR, gel electrophoresis) or UV-spectrophotometry. Using tissue sections, H&E and fluorescent DAPI stainings for quantifying DNA were done additionally.

**Results:**

DNase I-perfused aortas contained significantly lower amounts of dsDNA compared to native controls or detergent-perfused aortas. PicoGreen on tissue lysate showed 85.2% and 80.8% reduction in residual DNA; salted out DNA showed 90.3% and 84.6% reduction. Similarly, DAPI showed 91.1% and 82.4% reduction in DNA. QPCR reflected decreased concentration and fragmentation of residual DNA for salted out DNA, but not for solid-phase DNA. Gel electrophoresis using both purifications confirmed decreased DNA concentration and increased fragmentation, whereas spectrophotometry showed limited overall usability.

**Conclusion:**

Fluorescence-based DNA quantification using PicoGreen is useful for all three DNA purifications from decellularised aortas, and the only method applicable to tissue lysate. In turn, salted out DNA can be used reliably for PicoGreen, real-time QPCR and gel electrophoresis. Spectrophotometry is not recommended irrespective of the DNA purification. Complementary DAPI-based DNA quantification using tissue sections is advisable.

**Supplementary Information:**

The online version contains supplementary material available at 10.1007/s11033-025-10755-1.

## Introduction

Tissue engineering constitutes a promising tool to overcome shortages of available organ donors, which is an ever increasing issue given the worldwide population growth and increasing life span [1, 2]. Moreover, vascular diseases such as atherosclerosis, deep vein thrombosis or chronic venous insufficiency are globally distributed and require vascular replacement [3]. Using animal vessels for decellularisation and subsequent repopulation, a main strategy to engineer tissues or organs adapted to human needs, provides an excellent natural way to generate donor grafts. Such an approach complements bioprinting technologies and other synthetic approaches that are emerging [4, 5]. However, natural scaffolds have to be processed thoroughly to avoid graft rejection and inflammatory reactions after transplantation [6]. In addition, tissues rich in fibrous biomaterials like cartilage or aortae are difficult to decellularise or even resistant to decellularisation [7].

Decellularisation of extra-cellular matrix scaffolds such as from aortas [8] is usually achieved by physical, chemical and biological means [9, 10]. Despite perfusing small-diameter blood vessels of animal origin with the combined action of detergents (e.g. sodium dodecyl sulfate SDS, sodium deoxycholate SDC) and DNA-removing enzymes (e.g. DNase I) it is often challenging to achieve satisfactory decellularisation, i.e. removal of sufficient nuclear material, especially when applying protocols with a perfusion duration of less than 48 h [11, 12]. Nevertheless, thorough removal of nucleic acids, antigens and other cellular components, is essential in generating novel scaffolds to ensure their biocompatibility [13]. Immunological sensing of residual donor DNA in the graft may induce possible adverse reaction to xenotransplants in vivo [14, 15] Therefore, quantitative analysis of residual DNA is crucial to determine the efficiency of the decellularisation protocol and to estimate the potential risk of rejection of the engineered xenotransplant [13].

However, the methods used to purify DNA from decellularised tissue affect precision and usability for subsequent quantification of DNA. For example, silica-based solid-phase spin-columns that are widely used to purify DNA from decellularised tissue, do not completely purify small DNA fragments, which are often present in decellularised tissue [14, 16]. Thus, especially in decellularisation studies the amount of residual DNA is often underestimated, which in turn affects the potential risk for rejection after transplantation [14]. Other purification methods such as salting out DNA could overcome this issue [17]. Additionally, using tissue lysate directly for DNA quantification constitute a fast and precise and therefore promising alternative since additional DNA purification steps are not necessary, which avoids loss and thus underestimation of DNA content.

**Fig. 1:**
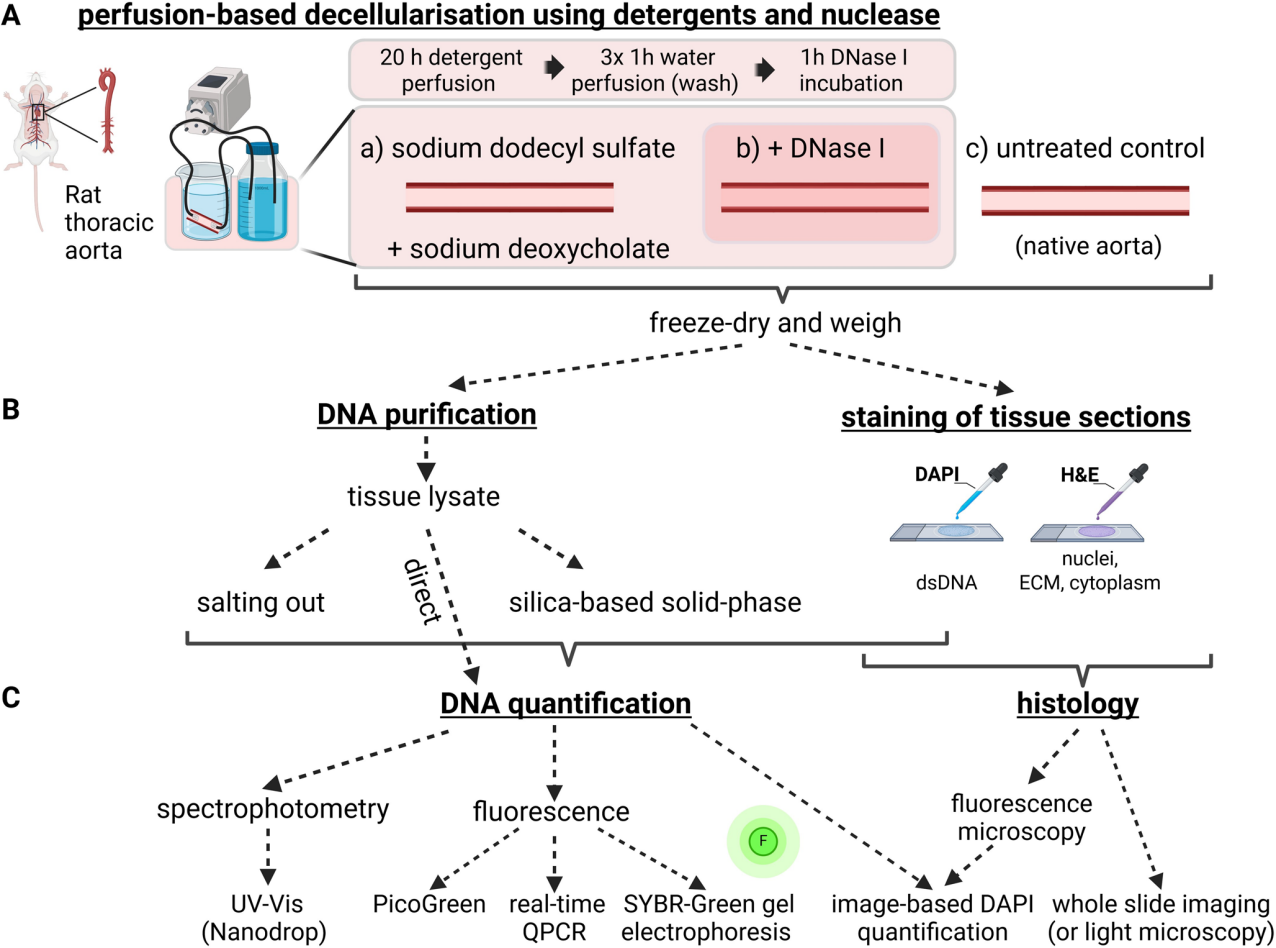
Overview and workflow of the methods used in this study. A Aortas were isolated from rats, perfused with SDS/SDC (1%), washed with deionized water and incubated with or without DNase I (50 U/ml). Untreated, native aortas were used as control. After freeze-drying and weighing, B decellularised aortas were digested by proteinase K (termed: tissue lysate) and directly used for DNA quantification; in addition, tissue lysate was further purified by salting out or by using silica-based solid-phase columns prior to quantify residual DNA. Furthermore, tissue sections were stained with H&E or DAPI. C DNA from the three purification methods was quantified and characterised by UV-Vis spectrophotometry (Nanodrop) or fluorescence-based methods (PicoGreen™, real-time QPCR, SYBR™ Green-based gel electrophoresis). Results were compared to histology-based characterization of residual DNA via image-based DAPI quantification and evaluation of whole slide imaging from H&E stained sections. Created in BioRender: https://BioRender.com/geu8bcc

In order to shed light on these issues, an exemplary perfusion-based decellularisation protocol completed within 24 h was developed for this study. It is based on the consecutive action of detergents (sodium dodecyl sulfate/sodium deoxycholate) and DNase I to perfuse thoracic aortas explanted from rats (Fig. 1A). It is hypothesized that residual DNA differs in its concentration and fragmentation after SDS/SDC-based perfusion with or without additional DNase I treatment. Moreover, it is tested which method for purifying DNA (tissue lysate, silica-based solid-phase, salting out) from decellularised rat aortas is most suited to measure and reflect the concentration of residual DNA after decellularisation. This is tested via different methods for DNA quantification including various fluorescence-based methods, spectrophotometry as well as histological stains including image-based quantification of DNA-binding dyes (Fig. 1B, C). In addition to above findings, this study did elucidate: (*i*) which method combination for DNA purification and quantification is most suited for characterising residual DNA in decellularised rat thoracic aortas; (*ii*) how tissue sections with fluorescent histological staining complement purification-based DNA quantification in evaluating the decellularisation efficiency of rat aortas; (*iii*) the impact of DNase I activity on the decellularisation efficiency in aortas.

## Materials and methods

### Extraction of blood vessels and sample sizes

Rat thoracic aortas (3–4 cm in length) were obtained from male animals (body weight was 410 ± 98 g) post-mortem as secondary use of cadavers from Wistar strains euthanized for other reasons (animal experiment licences and permits: UKJ-17-106, UKJ-22-036, twz02-2022, twz24-2023, twz04-2024). Extracted aortas were placed in a mixture of 5 ml phosphate-buffered saline and 1 ml dimethyl sulfoxide (Serva, 20385.02) and stored at -25 °C until further processing (see Table S1). Twenty-two rat aortas were extracted in total. Thereof, six were used as untreated and thus native control; eight were perfused with 1% SDS/SDC and eight other aortas were perfused with 1% SDS/SDC and additionally incubated with DNase I.

### Perfusion-based decellularisation

#### Decellularisation solution (1% SDS/SDC)

For preparing detergents to be used as perfusion-based decellularisation solution, 10 g sodium dodecyl sulfate (SDS, pellets, Carl Roth, CN30.2) and 10 g sodium deoxycholate (SDC, Sigma-Aldrich, D6750-100G) were dissolved in 1 l deionized water under stirring. This resulted in 1 l solution of 1% SDS with 1% SDC.

#### DNase I reaction solution

DNase I reaction solution was prepared as 25 mM MgCl_2_ (Sigma-Aldrich, M1028-1Ml), 100 mM Tris-HCl (Sigma-Aldrich, T2569-100ML) and 1 mM CaCl_2_ (Sigma-Aldrich, 21115-1ML) in deionized water including 50 units of DNase I (Invitrogen, 18047019) per ml reaction buffer.

#### Perfusion-based decellularisation

A peripheral venous cannula (type 18G, BD Angiocath, 381147) was inserted into each end of an aorta after the catheter was trimmed to a length of circa 1 cm (Fig. S1) to optimize perfusion through the aorta. Suture was wrapped around the ends of the aorta to ensure tight connection. Perfusion tubes were then connected to both cannulas and stabilized with medical tape. One free end was attached to the output side of a pump (Peristaltic pump, G728-1-1, Grothen), and the opposing end was placed into a 1 l glass bottle containing 1% SDS + SDC perfusion solution. Another perfusion tube was connected to the input side of the pump, and the free end was submerged in the solution. To prevent the aorta from drying out, it was placed into a small chamber and submerged in the perfusion solution. In general, the flow rate of the pump was set to 100 ml/min recirculating solution. SDS/SDC was perfused for 20 h at room temperature followed by perfusion with 1 l deionized water for 3 h at room temperature. The water was replaced every hour to efficiently remove residual SDS/SDC in the aorta (Figs. 1A, S1). Finally, 50 ml 1x DNase I buffer containing 2500 units of DNase I enzyme (= 50 U/ml) were perfused through the aorta for 1 h at 37 °C. Aortas lacking DNase I treatment were perfused only with DNase I buffer. As controls, native and untreated (i.e. non-perfused) aortas were used.

### DNA purification

#### Tissue lysate

Perfused aortas (SDS/SDC or SDS/SDC + DNase I) or untreated control aortas were freeze-dried by lyophilization using a VaCo2 device (Zirbus technology GmbH, Germany) and weighted with an electronic precision balance (Sartorius, AC 211 S). Aortas were transferred into a 1.5 ml tube containing 200 µl tissue digestion buffer (180 µl buffer ATL and 20 µl proteinase K, both from QIAamp DNA micro kit, Qiagen, 69504), pulse-vortexed for 15 s and incubated at 56℃ with shaking at 550 rpm in a thermomixer overnight to ensure full digestion of tissue. Tissue lysate was aliquoted and stored at -25℃ until further processing.

#### Salting out

Salting out of DNA followed stablished protocols [17–19]. In brief, 30 µl of tissue lysate were mixed with 90 µl of 2 M NaCl (Carl Roth, HN00.1), vortexed for 15s and centrifuged at 15,000 g for 10 min. The DNA-containing supernatant (pellet = protein debris) was transferred to a fresh 1.5 ml nuclease-free tube and the DNA was precipitated by adding ice-cold absolute ethanol (molecular biology grade, Fisher BioReagents, 16606002) at a 1:2 volume ratio. The mixture was centrifuged at 8000 g for 10 min, the supernatant was carefully removed and 500 µl of ice-cold 70% absolute ethanol were added to the pellet for washing. Following centrifugation for 10 min at 15,000 g, the supernatant was carefully removed and the DNA pellet was air-dried at 37℃ for 10 min with lid partly open. Finally, the DNA pellet was carefully dissolved in 30 µl nuclease-free water (Qiagen, 129114).

#### Silica-based solid-phase

The spin column-based QIAamp DNA micro kit (Qiagen, 69504, protocol: Isolation of Genomic DNA from Tissues) was used for silica-based solid-phase DNA purification. In brief, 30 µl of tissue lysate were mixed with 30 µl buffer AL. After vortexing for 15 s, 30 µl of 100% absolute ethanol were added, vortexed for 15 s and incubated for 5 min at room temperature. After a brief spin of the tube, the entire lysate was transferred to a QIAamp MinElute column and centrifuged at 6000 g for 1 min; the flow-through was discarded. Afterwards, 75 µl buffer AW1 were added into the QIAamp MinElute column tube and the mixture was centrifuged at 6000 g for 1 min. After the QIAamp MinElute column was transferred into a clean collection tube, 75 µl buffer AW2 were added and centrifuged at 6000 g for 1 min. For elution of DNA, QIAamp MinElute column was placed in a clean nuclease-free 1.5 ml tube and 30 µl nuclease-free water were applied to the centre of the membrane. The tube was incubated at room temperature for 1 min and centrifuged at 10,000 g for 3 min to elute the DNA.

### DNA quantification

Measuring concentration and fragmentation of DNA of the three above-mentioned purification methods from the three different treatments (native control, SDS/SDC-perfused, SDS/SDC + DNase I-perfused aortas) was carried out either via spectrophotometry or fluorescence (PicoGreen™, real-time QPCR, SYBR™ Green-based gel electrophoresis).

#### Spectrophotometry

DNA concentration was measured by UV-Vis spectrophotometry using Nanodrop1000 (Thermo Fisher). Absorbance-based quantification at 260 nm was used for quantifying DNA. Absorbance ratios (A_260_ /A_280_, A_260_ /A_230_) were measured for purity analysis. Two µl of purified DNA (tissue lysate undiluted and 10 times diluted; salted out undiluted and 10 times diluted; solid-phase undiluted) were measured. Nuclease-free water was used as blank.

#### Fluorescence assay using picogreen™

In order to determine DNA concentration by fluorescence, the Quant-iT™ PicoGreen™ dsDNA Assay-Kit (Invitrogen, P7589) including 20xTE buffer and lambda DNA standard was used. Standard and test samples were pipetted and measured in an opaque 96-well plate with white flat bottom (Nunc™ FluoroNunc™/LumiNunc™ 96-well plate, Thermo Scientific™, 437796). A standard curve was generated by diluting lambda DNA in 1xTE buffer (diluted from 20x using nuclease-free water) resulting in 1000, 500, 100 and 10 ng/ml including 0 ng/ml as blank. After diluting PicoGreen 200-fold in TE buffer, 100 µl of this working solution were mixed with (i) 100 µl of the different standard curve concentrations, or (ii) with 100 µl experimental DNA solution after mixing 1 µl purified sample DNA solution (obtained from tissue lysate, salting out or solid-phase purification) to a final volume of 100 µl in TE buffer. 200 µl of this assay solution were incubated per well in a 96-well plate for 5 min at room temperature in the dark. Fluorescence was measured using a microplate reader (Synergy LX, BioTek, Germany) using excitation at 480 nm and emission at 520 nm; three technical replicates were analysed. The fluorescence value of the blank was subtracted from that of each of sample; the DNA concentration of each sample was calculated based on the generated standard curve.

#### Real-time QPCR

Amplification reactions were set up in a total volume of 10 µl by mixing 5 µl SsoAdvanced™ Universal Probes Supermix (172–5281, Bio-Rad, including ROX as passive normalization dye) with 1 µl purified DNA solution (obtained from tissue lysate, salting out or solid-phase purification) as template. Salted out DNA was diluted 1:10 with nuclease-free water since a pilot experiment revealed that undiluted salted out DNA inhibited amplification and did not result in Cq values. As probe-based target primer 0.5 µl of HEX-labelled *Glyceraldehyde 3-phosphate dehydrogenase* (rat origin, unique assay ID: qHsaCEP0041396, PrimePCR™, Bio-Rad) and 3.5 µl nuclease-free water were added. Nuclease-free water instead of DNA template was used as negative control reaction. Reactions were thermo-cycled according to the following protocol: 3 min s at 95 °C, [(10 s at 95 °C, 25 s at 60 °C) x 40]. Fluorescent signals were measured on an AriaMx Real-time PCR system (Agilent Technologies) with a Cq normalized to ROX and set to a threshold of 0.022. Three technical replicates of one single biological sample with a Cq difference below 0.5 were averaged and used for calculating group (native, SDS/SDC-perfused, SDS/SDC + DNase I-perfused) mean values. Primer efficiency (98%) and coefficient of determination (R^2^ > 0.999) were verified and determined using a standard curve with five serial dilutions of DNA in nuclease-free water.

#### SYBR Green-based gel electrophoresis

To prepare a 1.0% gel 500 mg agarose (Agarose 1000 UltraPure, Invitrogen, 10975.035) were diluted into 50 ml 1x TAE buffer (diluted with 50x TAE buffer, Sigma- Aldrich, SRE0033-1 l), slowly heated in a microwave until brief boiling for 30 s and cooled down at room temperature for 5 min. After that, 5 µl of 10000x SYBR^®^ Green I nucleic gel stain (Cambrex, 50512) were added into the solution and carefully mixed. Finally, the solution was poured into a gel-casting tray with combs to solidify. One µl Tritrack DNA loading dye (Thermo Scientific, R1161) was mixed with 3 µl of each DNA sample. This mixture was loaded on the gel together with a 1 kb (GeneRuler 1 kb DNA ladder, ThermoScientific SM0312) and a 100 bp DNA size marker (GeneRuler 100 bp DNA ladder, ThermoScientific SM0241). Intact plasmid DNA (ptfLC3) with an approximate length of 6100 bp was loaded as positive control. Finally, the gel was run at 125 V for 1 h. The results were visualized by an UV transilluminator (Quantum 1000/26 M, Vilber Lourmat) equipped with Quantum-Capt software (version15.18, Vilber Lourmat).

### Histology

#### Tissue embedding and sectioning

For embedding of decellularised aortas (SDS/SDC-perfused or SDS/SDC + DNase I-perfused) and untreated controls, approximately 2 mm were cut before and after decellularisation, placed in an embedding cassette (Carl Roth, K114.1) and kept in 5% formaldehyde solution (Otto Fischar, 27237) at 4 °C for 12 h. Dehydration of tissue was done by incubation in an ascending alcohol series of 70%, 95% and 100% ethanol as well as in 100% xylene (Roth, 9713.5) using an automatic tissue processor (TP1020, Leica). Embedding of tissues in paraffin (HistoSec™, Merck, 1.11609) was done using a tissue embedding machine (HistoCore Arcadia H&C, Leica). Tissue blocks were cut into 3 μm thin sections using an automatic rotary microtome (HM 355 S, FisherScientific). Sections were placed on microscope slides (SuperFrost^®^Plus, J1800AMNZ, ThermoScientific) and incubated at 60 °C for 1 h to improve the adhesion of sectioned aortas to the glass surface.

#### Hematoxylin and eosin (H&E) staining

Hematoxylin solution was prepared by dissolving 15 g potassium aluminium sulfate (Roth, P724.2) and 300 mg hematoxylin (Roth, 3816.1) in 300 ml deionized water under stirring. Subsequently, 60 mg sodium iodate (Sigma, S-4007) and 15 g chloral hydrate (Roth, K318.1) were added under stirring. The pH was adjusted to around 2.5 by adding citric acid (Roth, 3958.2). The solution was heated to boiling and was cooled down. Slides with tissue sections were rehydrated by immersion in a descending alcohol series (xylene; 100%, 95% and 70% ethanol) twice for 2 min each. Slides were incubated with hematoxylin for 5 min and then washed with water for 10 min. Afterwards, the slides were incubated with eosin Y solution (Sigma-Aldrich, HT110132) for 1 min. Slides were dehydrated with 70% ethanol, 96% ethanol, 100% ethanol and xylene, using two incubations for 2 min each. Finally, slides were mounted with CV mount (Leica, 1406430011), covered with a cover slip and scanned with a NanoZoomer 2.0-HT slide scanner (Hamamatsu Photonics). NDPview2 image viewing software was used to display and analyse the images (Hamamatsu).

#### DAPI staining and image-based DNA quantification

Fluorescence solution was generated by mixing citrate phosphate buffer (100 mM citric acid, 200 mM Na_2_HPO_4_, pH 7.0) with DAPI (4′,6-Diamidin-2-phenylindol-dihydrochlorid; Sigma, D9542-1MG) to a final concentration of 0.2 ng/µl to visualize nuclear dsDNA. Tissue slides were rehydrated (see above ), incubated in citrate phosphate buffer for 5 min and stained in fluorescence solution for 30 min in the dark. Slides were mounted with anti-fade Fluorescence Mounting Medium (Abcam, ab104135). Fluorescence microscopy (Axio Imager M1, Zeiss) was used to visualize fluorescent DAPI signals at Ex/Em = 364 /454 nm. Fluorescent and brightfield images were acquired by Zen Pro software (Zeiss) and loaded in Fiji [20] for image-based quantification of residual DNA in DAPI-stained sections using a Python code [21]. In brief, images were cropped by hand to show tissue and DAPI signal; each image was analysed with a Python code to calculate the relative surface covered by nuclear DNA material according to the equation: *P* = I*100/B (I is the number of pixels of the nuclei, B is the number of pixels of the sectioned aorta). The Python code reads the input images and creates an output with information given as relative surface of stained tissue section in percent.

### Statistical analysis

Group comparisons (i.e. native controls versus SDS/SDC*-*perfused versus SDS/SDC + DNase I-perfused aortas) were calculated for differences in residual DNA content for each purification method (tissue lysate, salting out, solid-phase) and each quantification method (spectrophotometry, PicoGreen, QPCR, image-based DAPI) by One-Way ANOVA with Tukey-HSD as post-hoc test. Normal distribution of data was checked and verified by Shapiro-Wilk test. In all cases, SPSS 29 (IBM) was used to calculate statistical analyses, while Interactive Dotplot was used for visualising data as Box plots [22].

## Results

Results from Sect. 3.1 to 3.4 are summarized in supplementary material S2.

### DNA quantification by UV-Vis spectrophotometry

**Fig. 2:**
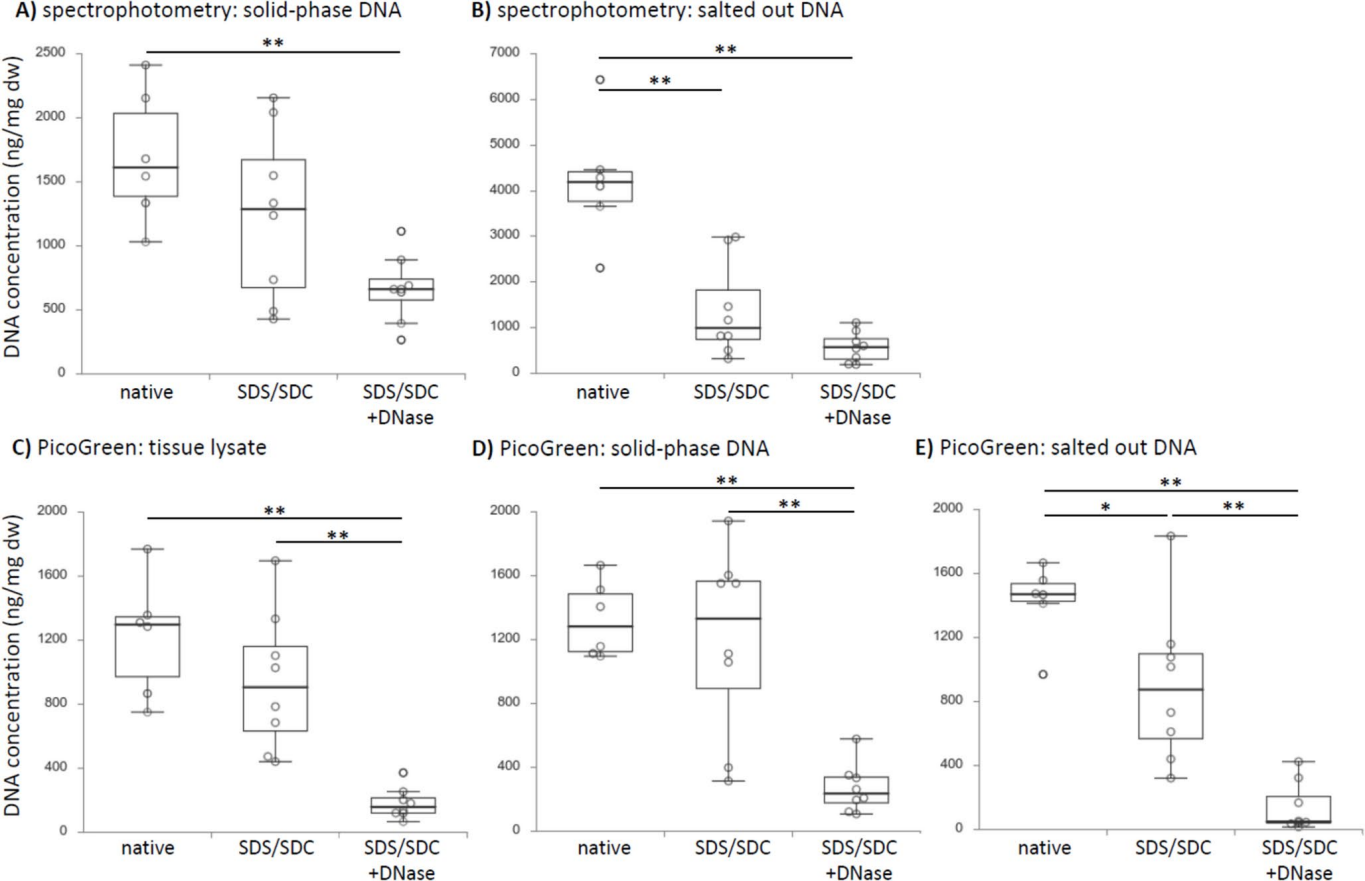
Spectrophotometric and fluorescent quantification of residual DNA purified by different methods from aortas after perfusion with 1% SDS/SDC ± DNase I. DNA was purified by means of silica-based solid-phase, salting out or using the tissue lysate directly. Nanodrop was used to measure DNA concentration by UV-Vis spectrophotometry of DNA purified by silica-based solid-phase A or salted out DNA B. Note that measuring tissue lysate did not allow meaningful DNA quantification, even not when diluted in nuclease-free water ten times, probably because purity ratios were very low due to protein contamination. DNA quantification using the dsDNA-binding fluorescent dye PicoGreen of tissue lysates C, or of DNA purified by solid-phase D or salted out DNA E. * indicates significant differences between the respective groups with p < 0.05; ** indicates p ≤ 0.001

Measuring solid-phase purified DNA using Nanodrop spectrophotometry did not show significant differences between the mean DNA content of native aortas (1692 ± 191 s.e.m. ng/mg dry weight dw) and SDS/SDC-perfused aortas (1246 ± 219 s.e.m. ng/mg dw) (Fig. 2A). This equalled a decrease of 26.4%. However, there was a highly significant (*p* = 0.004) reduction of SDS/SDC + DNase I-perfused aortas (665 ± 87 s.e.m. ng/mg dw) compared to native aortas. This equalled a decrease of 60.7%. Decrease in residual DNA comparing SDS/SDC and SDS/SDC + DNase I-perfused aortas was 46.7%. Measuring salted out purified DNA by Nanodrop showed the highest mean concentration for native aortas (4207 ± 498 s.e.m. ng/mg dw) and a highly significant (*p* < 0.001) decrease in SDS/SDC-perfused aortas (1375 ± 343 s.e.m. ng/mg dw). A further decrease in residual DNA content was found in SDS/SDC + DNase I-perfused aortas (579 ± 109 s.e.m. ng/mg dw), representing a highly significant difference (*p* < 0.001) compared to native aortas (Fig. 2B). This equalled a mean reduction of residual DNA in SDS/SDC and SDS/SDC + DNase I-perfused aortas of 67.3% and 86.2% compared to native aortas.

DNA concentration in tissue lysates using Nanodrop was not evaluable neither for undiluted nor for diluted lysates due to very low mean values of purity ratios (A_260/280_: 0.931 ± 0.005 s.e.m. for diluted samples, 0.885 ± 0.018 s.e.m. for undiluted samples; A_260/230_: 0.183 ± 0.016 s.e.m. for diluted samples; -0.315 ± 0.385 s.e.m. for undiluted samples).

### DNA quantification by PicoGreen

PicoGreen™-based DNA quantification using the tissue lysate directly showed highest mean values in native aortas (1223 ± 137.2 s.e.m. ng/mg dw) and a decrease of 22.9% compared to SDS/SDC perfused aortas (943.1 ± 143.3 s.e.m. ng/mg dw) (Fig. 2C). DNA concentration in tissue lysates of SDS/SDC + DNase I-perfused aortas (181.4 ± 36.6 s.e.m. ng/mg dw) was highly significantly lower compared to native aortas (*p* < 0.001) and to SDS/SDC-perfused aortas (*p* = 0.001). This equalled a decrease in DNA concentration by 85.2% (comparing SDS/SDC + DNase I-perfused with native aortas) and 80.8% (comparing SDS/SDC + DNase I-perfused with SDS/SDC-perfused aortas), respectively. Solid-phase purified DNA showed a difference of 10.1% in mean DNA concentration between native aortas (1324.9 ± 88.5 s.e.m. ng/mg dw) and SDS/SDC-perfused aortas (1191.7 ± 194.2 s.e.m. ng/mg dw) (Fig. 2D). DNA of SDS/SDC + DNase I-perfused aortas measured with PicoGreen and purified by solid-phase (269.8 ± 53.9 s.e.m. ng/mg dw) was highly significantly lower than native aortas (*p* < 0.001) and highly significantly lower than SDS/SDC-perfused aortas (*p* < 0.001). This equalled a decrease in DNA concentration by 79.6% (comparing SDS/SDC + DNase I-perfused with native aortas) and 77.4% (comparing SDS/SDC + DNase I-perfused with SDS/SDC-perfused aortas), respectively. Salted out purified DNA from native aortas had a mean concentration of 1424.8 ± 89.4 s.e.m. ng/mg dw, whereas SDS/SDC-perfused aortas had 36.9% less DNA (898.8 ± 160.2 s.e.m. ng/mg dw). This difference was statistically significant (*p* = 0.022). SDS/SDC + DNase I-perfused aortas had 90.3% less DNA (138.2 ± 55.2 s.e.m. ng/mg dw) than native controls and 84.6% less than SDS/SDC-perfused aortas (Fig. 2E). These differences were highly significant from each other (both *p* < 0.001).

### Image-based DNA quantification using DAPI

**Fig. 3:**
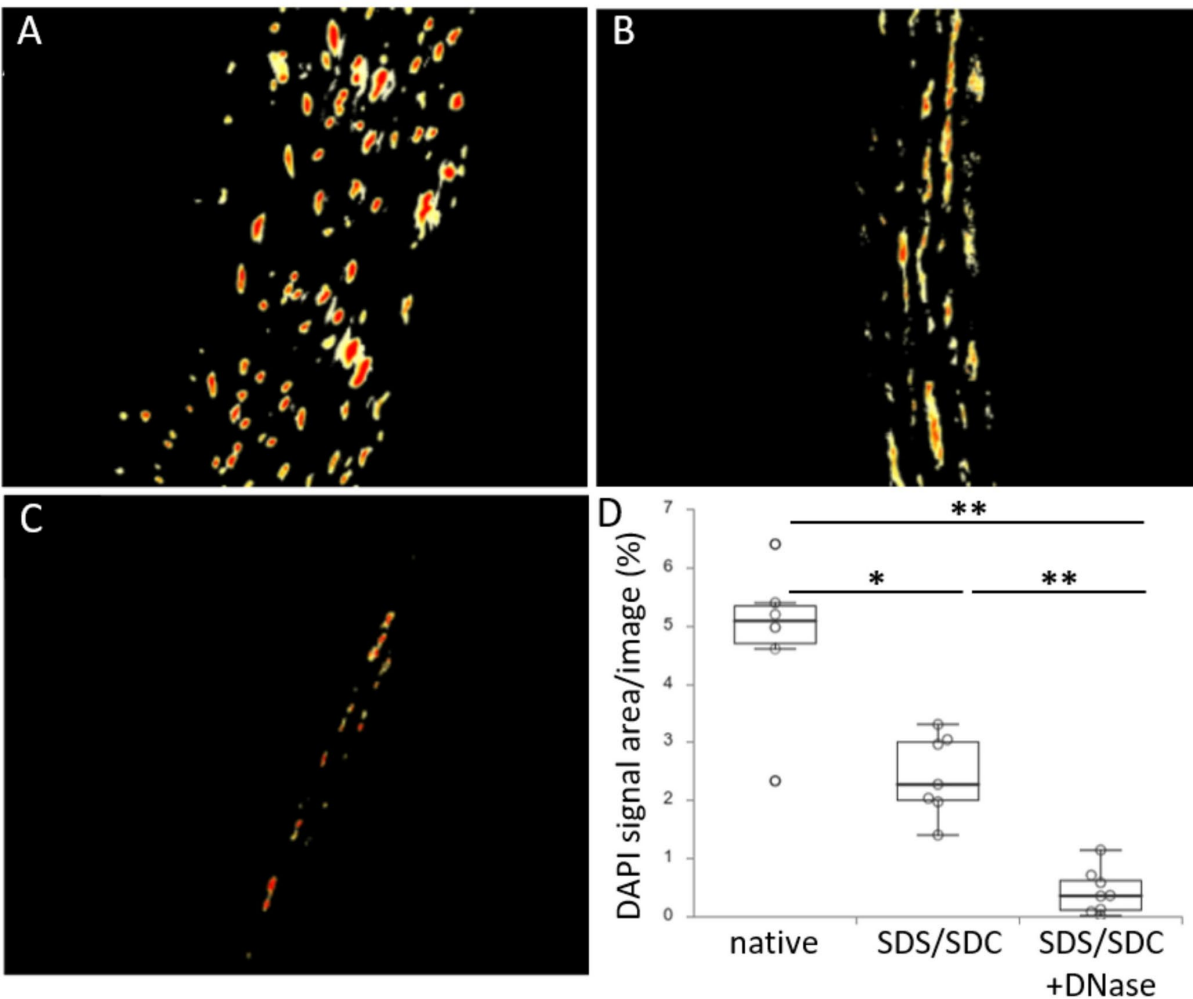
DNA quantification using images from DAPI-stained tissue sections. Representative images from tissue sections of decellularised rat aortas stained with DAPI, visualised by fluorescence microscopy and measurement of the signal area. A Native rat aorta. B Aorta perfused with 1% SDS/SDC. C Aorta perfused with 1% SDS/SDC followed by DNase I incubation. D Quantification of DAPI signal intensity from images of each treatment; * indicates significant differences between the respective groups with p < 0.05; ** indicates p ≤ 0.001

Image-based DNA quantification using tissue sections stained with DAPI showed highest relative DAPI signal in native aortas (mean 4.82% ± 0.55 s.e.m. stained area per total area of the section, Fig. 3A). SDS/SDC-perfused aortas showed a significant (*p* = 0.003) lower signal of DAPI (mean 2.43% ± 0.26 s.e.m.) indicating a decrease by 49.5% (Fig. 3B). SDS/SDC + DNase I-perfused aortas showed a mean signal of 0.43% ± 0.13 s.e.m., which is highly significantly lower than native aortas and SDS/SDC-perfused aortas (both *p* values < 0.001) (Fig. 3C, D). This equalled a decrease in DAPI signal and thus DNA concentration by 91.1% and 82.4%, respectively (Fig. 3D).

### DNA quantification by QPCR

**Fig. 4:**
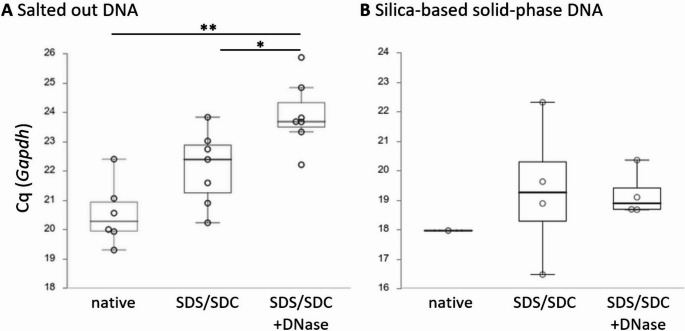
Mean QPCR-derived Cq values of DNA purified from aortas perfused with SDS/SDC ± DNase I. After decellularising rat aortas with 1% SDS/SDC or 1% SDS/SDC followed by DNase I incubation, template DNA was purified by A salting out, or B silica-based solid-phase, and used for real-time QPCR with Gapdh as marker gene. Salted out DNA was ten times diluted with nuclease-free water before running QPCR. Low Cq values indicate a relative high concentration (and low degree of fragmentation) of DNA, whereas high Cq values indicate relative low concentration (and high degree of fragmentation) of DNA. Note that no Cq values could be obtained from tissue lysate, even not when diluted ten times in water. * indicates significant differences between the respective groups with p < 0.05; ** indicates p ≤ 0.001

Cq values determined by real-time PCR targeting the house keeping gene *Gapdh* increased when salted out DNA was analysed from native over SDS/SDC to SDS/SDC + DNase I-perfused aortas (Fig. 4A). Mean Cq values derived from DNA purified by salting out for each of the three groups were 20.54 (± 0.44 s.e.m.) for native DNA, 22.10 (± 0.48 s.e.m.) for SDS/SDC-perfused aortas and 23.92 (± 0.44 s.e.m.) for SDS/SDC + DNase I-perfused aorta DNA. Highly significant differences were found between native and SDS/SDC + DNase I-perfused aortas (*p* < 0.001). Group difference between SDS/SDC and SDS/SDC + DNase I-perfused aorta DNA was significant (*p* = 0.013). When silica-based solid-phase purified DNA was used for the same QPCR-based analysis (Fig. 4B), reliable Cq values could only be obtained from a limited number of biological replicates (native: one out of six; SDS/SDC-perfused: four out of eight; SDS/SDC + DNase I-perfused: for out of seven) due to a lack of amplification or because technical replicates differed strongly (> 1.5). From the samples that resulted in Cq values, mean values from solid-phase purified DNA were 17.97 for native DNA, 19.33 (± 1.20 s.e.m.) for SDS/SDC-perfused aortas and 19.21 (± 0.40 s.e.m.) for SDS/SDC + DNase I-perfused aorta DNA. No Cq values could be obtained from tissue lysate, neither from native nor from SDS/SDC or SDS/SDC + DNase I-perfused aortas. Thus, no PCR-based amplification was possible in these samples even not when tissue lysate was diluted 1:10 with nuclease-free water.

### Gel electrophoresis

**Fig. 5:**
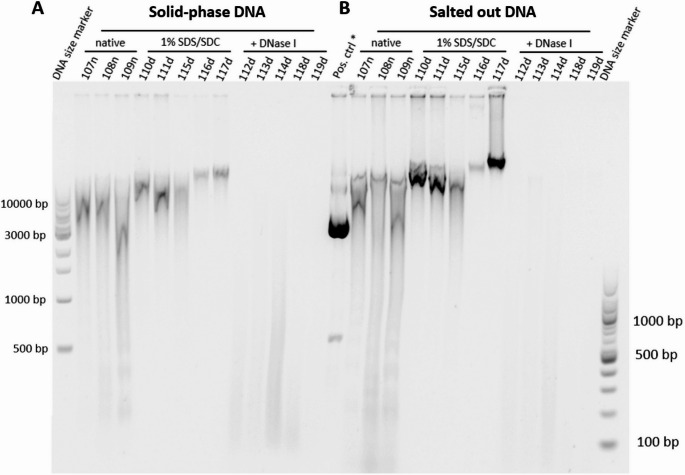
Gel electrophoresis of DNA purified by different methods from aortas perfused with SDS/SDC ± DNase I. Fluorescent bands of DNA purified from native aortas or those that were perfused with 1% SDS/SDC or perfused with 1% SDS/SDC followed by DNase I incubation by means of A silica-based solid-phase or B salting out DNA purification. Representative biological samples are labelled as three-digit number at top of gel; *As positive control intact plasmid DNA was used.

Gel electrophoresis analysis using SYBR Green-based dsDNA staining indicated an increasing degradation and fragmentation from native over SDS/SDC-perfused to SDS/SDC + DNase I-perfused aorta DNA. This was found for DNA that was purified by solid-phase and especially by salting out (Fig. 5A, B). As seen on the fluorescent agarose gel, native DNA showed intense bands (often in smears) at sizes above 10 kb length, while SDS/SDC-perfused aorta DNA appeared similar with a tendency of less intense bands. In lanes of SDS/SDC + DNase I-perfused aorta DNA either no bands were visible for several samples or only slight, hardly visible smears at reduced fragment size (circa 100–1000 bp) appeared. These results indicate strongly reduced amounts and fragment lengths of residual DNA for SDS/SDC + DNase I-perfused aorta DNA, which was found for DNA purified by salting out and by solid-phase silica-based method.

### Histology

**Fig. 6:**
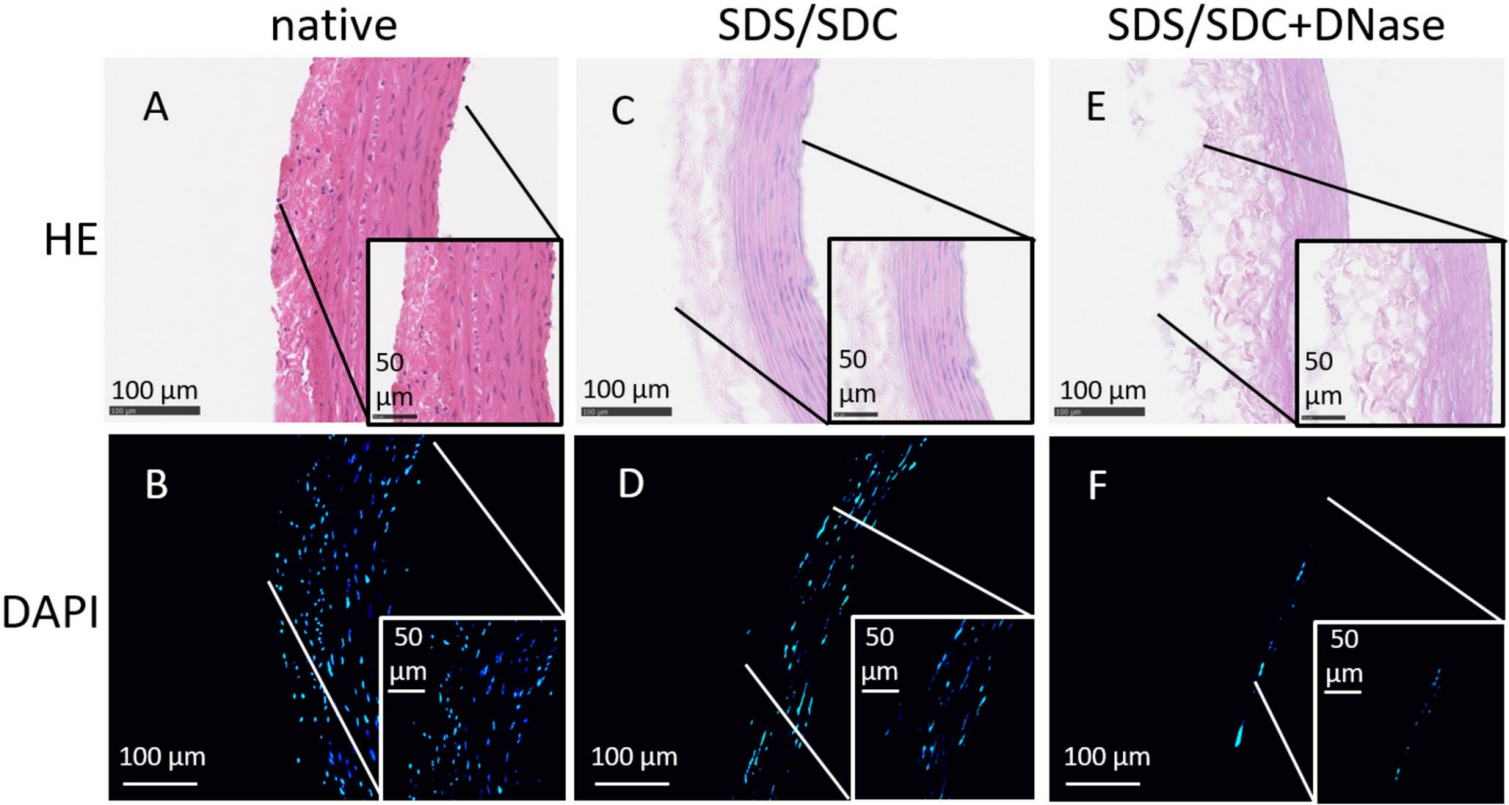
Stained tissue sections of rat aortas perfused with SDS/SDC ± DNase I. A Native and untreated aortas showed numerous nuclei with DNA as indicated by H&E staining (dark blue dots), and B DAPI staining (light blue dots). C, D Aortas perfused with 1% SDS/SDC showed reduced density of nuclei, E, F which was further reduced when aortas were perfused in addition with DNase I (50 U/ml).

Histological stains of sectioned native aortas showed a rather loose, but homogenous distribution of nuclei and thus DNA when using H&E (Fig. 6A) or fluorescent DAPI (Fig. 6B) as indicated by dark blue and light blue dots, respectively. Stains of SDS/SDC-perfused aortas showed a reduction in the number of nuclei with almost no remaining nuclei according to H&E (Fig. 6C), whereas a number of nuclei was stained by DAPI (Fig. 6D). Additional DNase treatment after SDS/SDC perfusion resulted in further decrease and almost no traces of nuclear DNA according to H&E (Fig. 6E) and DAPI signal intensity (Fig. 6F).

## Discussion

Decellularized tissues are gaining importance in regenerative medicine to address the demand for transplants. Ensuring their quality, especially by evaluating residual DNA, is critical but technically challenging. Therefore, our study compared and elucidated various methods to purify and determine DNA content in decellularised aortas. In particular, we revealed that residual DNA can be quantified using fluorescent dyes (e.g. PicoGreen) directly on tissue lysates, i.e. after proteinase-based digestion of tissue. This finding complements another recent study using decellularized skeletal muscle from porcine [16]. In addition, we have shown that DNA purification by salting out enables using complementary methods (e.g. real-time PCR) to quantify and characterise residual DNA. In contrast, DNA purification using silica-based solid-phase turned out to have limitations (especially with respect to PCR) which is in line with other studies [14, 16]. When spatial information of residual DNA in decellularised tissue is required, histological staining with H&E and DAPI is recommended since both can serve as quality control: while H&E staining is suitable to gain morphological information, DAPI is suitable for an image-based quantification of residual DNA in tissue sections. Moreover, to remove DNA from thick-wall tissues such as aortas efficiently, the use of DNase I after detergent-based perfusion is recommended, since it leads to a significant reduction of residual DNA. In combination with the above-mentioned methods for purifying and quantifying DNA, our results provide the basis to assess residual DNA in decellularised tissues to minimize the risk of adverse reactions for e.g. tissue engineering and transplantation purposes.

**Fig. 7:**
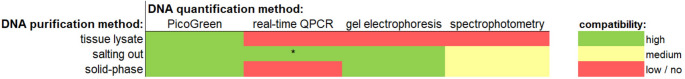
Compatibility of three DNA purification methods from decellularised rat aortas with four different DNA quantification methods. In addition, residual DNA can also be quantified by image-based analysis using DAPI-stained tissue sections (see: Image-based DNA quantification using DAPI). *template DNA was diluted 1:10 in nuclease-free water

The combination of ionic detergents and nuclease to achieve removal of cellular components during decellularisation is a common and efficient strategy in tissue engineering and used for different tissues and organs [10, 23]. This is confirmed in our study using rat aortas that were perfused with 1%SDS/SDC followed by DNase I (50 U/ml) incubation. Since residual DNA is regarded as a potent immunogen and indirect index of remaining cellular materials [23, 24], quantification of residual DNA is essential in decellularisation studies that aim for in vivo applications. As shown in our study, a strong decrease in residual DNA can be achieved via a short (24 h) perfusion-based protocol and be tracked via cost-efficient methods for purification and quantification. Moreover, given that tissues rich in fibrous biomaterials like rat thoracic aortas are difficult to decellularise or even considered as resistant to decellularisation [7, 25], our procedure may serve as template for decellularisation for such difficult-to-decellularise tissues. Our study also revealed that quantification of residual DNA in decellularised aortae is affected by the method used for DNA purification and quantification. This fact is often neglected in decellularisation studies with potentially severe consequences for clinical use such as in the case of xenotransplantation [14]. This renders precise measurement of residual DNA crucial for evaluating suitability of decellularized tissue. Thus, our study sheds valuable insights into these issues by highlighting three main findings that elucidated the optimal combination of methods for purification and quantification of DNA after tissue decellularisation:

(i) DNA from aortas digested by proteinase K (i.e. tissue lysate) can be directly quantified using PicoGreen without the need for further purification. This is time-and cost-efficient, whereas other quantification methods were not compatible with tissue lysate (Fig. 7) - most likely, because the high content of proteins and cellular debris inhibited real-time PCR or resulted in overloaded bands during gel electrophoresis and high background in spectrophotometry. Thus, using PicoGreen for direct quantification of residual DNA in proteinase-digested aortas that were decellularised confirms the usability of this dsDNA-binding fluorescent dye in decellularisation studies and therefore complements other studies [14, 16, 26] using similar fluorescent methods but other tissue sources. Moreover, similar to QPCR, PicoGreen reflects the degree of DNA fragmentation with a decrease in measured concentration indicating high degree of DNA fragmentation [27]. Our PicoGreen results also show that fluorescence-based dsDNA quantification is more reliable than UV-Vis spectrophotometry [28], which is often used to measure concentration of DNA purified from decellularised tissue [14]. However, despite its speed and accuracy for pure DNA, spectrophotometry measures all types of nucleotides in solutions containing DNA. Given a decreased concentration, but increased fragmentation of residual DNA especially in decellularised tissue, this technique shows less precision in measuring DNA purified from decellularised tissue.
(ii) DNA purification from decellularised aortas by salting out itself is not only convenient and cost-efficient, but also allows broad usability and flexibility with respect to different quantification methods. As our results have shown, salted out DNA can be used for quantification by PicoGreen, fragment visualisation in gel electrophoresis and real-time PCR (Fig. 7). Noticeably, salting out was the only DNA purification method that could be used for quantification by real-time PCR. One reason could be the loss of short fragments, including the target sequence *Gapdh*, when silica-based solid-phase purification was used [14, 29]. This issue resulted in inconsistent or even lack of real-time PCR results. Application of salted out DNA for UV-Vis spectrophotometry was possible, but of limited usability (Fig. 7), probably because remaining salt led to overestimation of DNA especially in native tissue (similar to 30). In case of decellularisation, this issue leads to an overestimation of total DNA reduction in decellularised tissue, which in turn could compromise tissue safety for in vivo applications.
(iii) Perfusing aortas with DNase I (after detergent-based perfusion and washing) results in strongly reduced content of residual DNA in comparison to native aortas or using detergents only. This was found for all quantification methods that showed at least high or medium compatibility with the according purification method (Fig. 7). Thus, DNase I activity has a significant impact on the efficiency of perfusion-based decellularisation in rat thoracic aortae. This finding confirms the results of another study using porcine aortas showing a high impact of DNase I on the decellularisation efficiency even though concentration and incubations time were different from our study [7]. Moreover, since DNase I activity usually results not only in elimination of DNA, especially in combination with a perfusion-based protocol, but also in degradation of DNA, methods used for quantifying residual DNA should ideally be able to quantify both concentration and fragmentation of DNA. Using salted out DNA and QPCR complemented by agarose gel electrophoresis as done in this study is an approach towards reaching this goal [28, 31], which could further be complemented by automated electrophoresis and digital fragment analysis for increased precision.

Using the combination of detergents and DNase to perfuse rat thoracic aortas led to a reduction of 90.3% and 91.1% in residual DNA as measured by PicoGreen on salted out DNA and by DAPI, respectively (Figs. 2B and 3). Such reduction in residual DNA is in the range found by other studies stating DNA reduction from 85 to 95% [32–35]. Despite the high reduction in total DNA amount, the lowest mean group value in our study was 138.2 ng DNA per mg dw using salted out DNA measured by PicoGreen. This value is above 50 ng/mg dw, which is suggested as a requirement for decellularisation among other parameters [10]. However, there is a number of studies showing that “decellularised” tissue still contain considerable amounts of residual DNA that are above this threshold. For example, porcine auricular cartilage at a certain size contained above 100 ng DNA per mg dw even after one week of DNase treatment [36] while the lowest DNA amount in porcine notochordal cell-derived matrices was approximately 85 ng per mg dw [32]. Similarly, decellularised porcine aorta had at least an average residual DNA amount of over 165 ng (expressed per wet weight) [25] which corresponds to at least  500 ng/mg dry weight. This is in line with the suggestions of other studies that no decellularisation process can achieve complete removal of cellular material, i.e. a certain amount of residual DNA will remain in most tested tissues [37]. Similarly, almost every tenth decellularisation study showed that DNA reduction was below 50% only [38]. Moreover, next to concentration, DNA fragment length is an important indicator of how effectively nucleic material has been removed or degraded during decellularisation; usually fragments below 200 bp satisfy the intent of decellularisation [35]. DNA of DNase I-perfused aortas showed fragment lengths below and above this value and requires further optimisation as indicated below. Nevertheless, gel electrophoresis confirmed a strong overall decrease of DNA content as well as reduction in fragment length compared to DNA from native and detergent-perfused aortas. Thus, even though detergents and mainly DNase eliminate and degrade the majority of DNA as shown in this study, other cellular components may still exist in the “decellularised” tissue or scaffold [39]. Surprisingly, clinical application of certain commercially available biomaterials, which contain more than 50 ng/mg of residual DNA, have shown good tolerance [40]. Nevertheless, to further reduce residual DNA content and fragment length in decellularised aortas and for translational reasons, longer incubation times which DNase I (e.g. 2 × 3 h) or higher concentrations of DNase I (e.g. 100 instead of 50 U/ml) are recommended in future studies.

Histological evaluation of our SDS/SDC-perfused aortas showed a strong decrease in DAPI signal and thus nuclear DNA content in comparison to native aortas with almost no DAPI signal left in tissue sections of SDS/SDC + DNase I-perfused aortas (Figs. 3 and 6). These results are similar to other detergent-based protocols complemented via DNase I application for decellularisation of animal aortas [7, 41, 42]. Absence of visible nuclear material in tissue sections stained with DAPI is another important parameter to account tissue as decellularised [10]. Therefore, as achieved in this study, a multiprocedural analysis including efficient methods for DNA purification and fluorescence-based quantification of residual DNA including histological sections [43] constitute the basis for reliable characterization of residual DNA in decellularised aortas.

## Electronic supplementary material

Below is the link to the electronic supplementary material.

Supplementary Material 1

Supplementary Material 2

## Data Availability

All data supporting the findings of this study are available within the paper and its Supplementary Information.
