# Supplementary material for: Determination of residual DNA in decellularised aortas– towards fluorescence-based quantification of DNA purified by various methods
Source: Mol Biol Rep. 2025 Jul 8;52(1):682. doi: 10.1007/s11033-025-10755-1 (PMC12238089; doi:10.1007/s11033-025-10755-1)

**Supplementary Table S1.** Basic information of rat thoracic aortas used for decellularisation in this study.

| **species / strain** | **sample ID** | **animal ID** | **treatment** |
| --- | --- | --- | --- |
| Rat / wistar | BVD-095 | TRANS-00090 | **native (untreated)** |
| Rat / wistar | BVD-096 | TRANS-00071 | native (untreated) |
| Rat / wistar | BVD-097 | TRANS-00097 | native (untreated) |
| Rat / wistar | BVD-107 | TRANS-00076 | native (untreated) |
| Rat / wistar | BVD-108 | TRANS-00123 | native (untreated) |
| Rat / wistar | BVD-109 | TRANS-00124 | native (untreated) |
| Rat / wistar | BVD-099 | TRANS-00092 | **perfusion with 1% SDS/SDC** |
| Rat / wistar | BVD-100 | TRANS-00088 | perfusion with 1% SDS/SDC |
| Rat / wistar | BVD-105 | TRANS-00039 | perfusion with 1% SDS/SDC |
| Rat / wistar | BVD-110 | TRANS-00049 | perfusion with 1% SDS/SDC |
| Rat / wistar | BVD-111 | TRANS-00037 | perfusion with 1% SDS/SDC |
| Rat / wistar | BVD-115 | TRANS-00073 | perfusion with 1% SDS/SDC |
| Rat / wistar | BVD-116 | TRANS-00325 | perfusion with 1% SDS/SDC |
| Rat / wistar | BVD-117 | TRANS-00324 | perfusion with 1% SDS/SDC |
| Rat / wistar | BVD-102 | TRANS-00119 | **perfusion with 1% SDS/SDC + DNase I (50 U/ml)** |
| Rat / wistar | BVD-103 | TRANS-00063 | perfusion with 1% SDS/SDC + DNase I (50 U/ml) |
| Rat / wistar | BVD-106 | TRANS-00091 | perfusion with 1% SDS/SDC + DNase I (50 U/ml) |
| Rat / wistar | BVD-112 | TRANS-00072 | perfusion with 1% SDS/SDC + DNase I (50 U/ml) |
| Rat / wistar | BVD-113 | BIOCH-67731 | perfusion with 1% SDS/SDC + DNase I (50 U/ml) |
| Rat / wistar | BVD-114 | TRANS-00075 | perfusion with 1% SDS/SDC + DNase I (50 U/ml) |
| Rat / wistar | BVD-118 | TRANS-00328 | perfusion with 1% SDS/SDC + DNase I (50 U/ml) |
| Rat / wistar | BVD-119 | TRANS-00335 | perfusion with 1% SDS/SDC + DNase I (50 U/ml) |

**Supplementary Figure S1.** Preparation of the perfusion system used for decellularisation. Created in BioRender: <https://BioRender.com/hld4221>


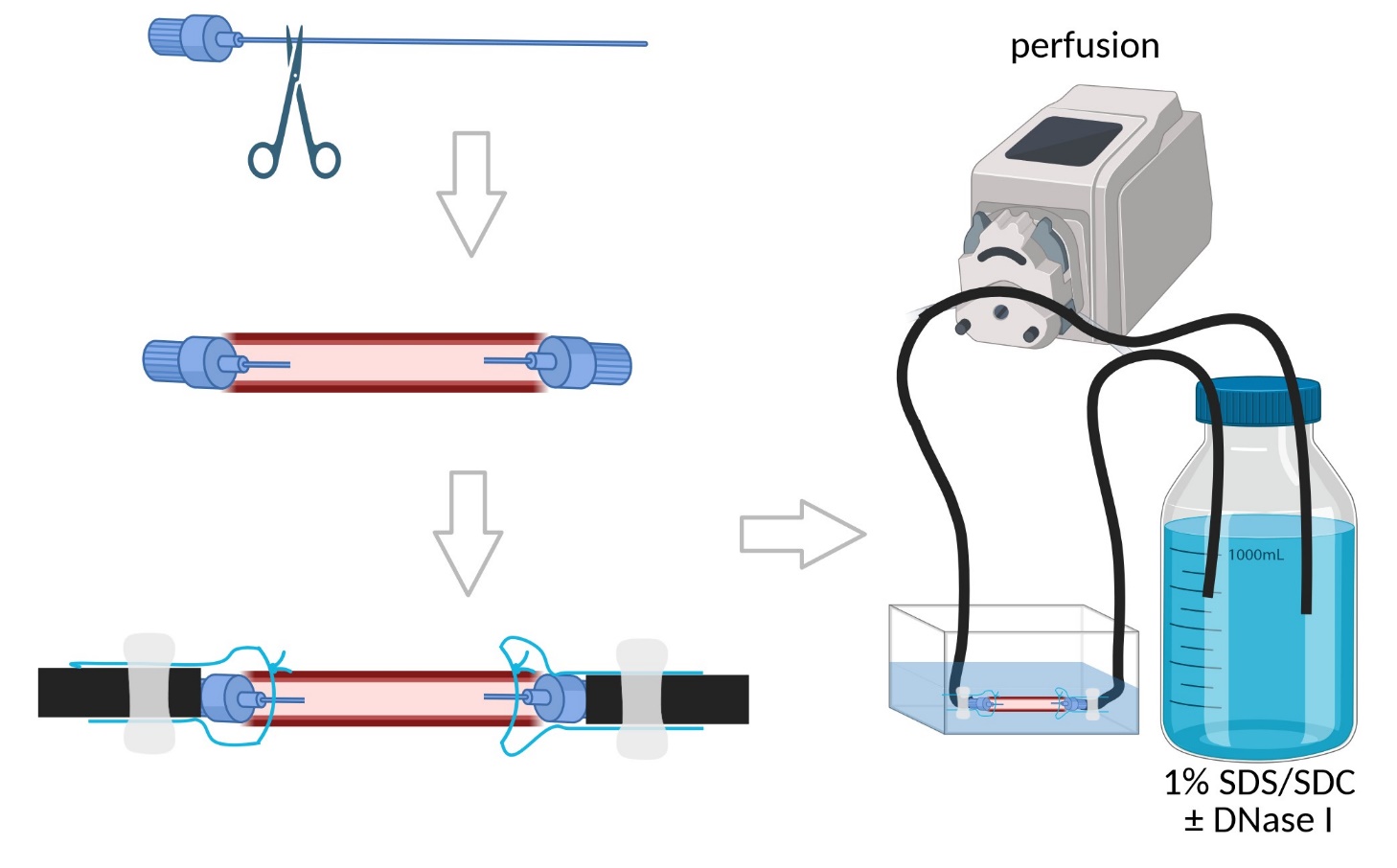

Supplement: Supplementary file 1 — Supplementary Material 1 [file 11033_2025_10755_MOESM1_ESM.docx]
