# Supplementary material for: Determination of residual DNA in decellularised aortas– towards fluorescence-based quantification of DNA purified by various methods
Source: Mol Biol Rep. 2025 Jul 8;52(1):682. doi: 10.1007/s11033-025-10755-1 (PMC12238089; doi:10.1007/s11033-025-10755-1)
Supplement: Supplementary file 2 — Supplementary Material 2 [file 11033_2025_10755_MOESM2_ESM.docx]

**Supplementary Table S2**. Summary of the DNA quantification results using different methods for purification and analysis after decellularisation of rat thoracic aortas by detergents (SDS/SDC) or additional treatment with DNase I; native and untreated aortas were used as control. Values represent mean values ± s.e.m. in ng DNA / mg dry weight of tissue. For QPCR measurements, values indicate Cq values; accordingly difference in Cq is given instead of decrease in %. For image-based DNA quantification using DAPI, values indicate the relative surface of stained tissue section in %. *Only a limited number of biological replicates resulted in Cq values. *Abbreviations*: UV-Vis – UV-visible spectrophotometry (Nanodrop); n.d. – not detected; n.s. – not significant.

|  |  |  |  |  |  |  |  |  |  |  |  |
| --- | --- | --- | --- | --- | --- | --- | --- | --- | --- | --- | --- |
|  |  | **Decellularisation method** | | | | **Statistics** | | | | | |
| **Analysis method** | **Purification method** | native  control | SDS/SDC | | SDS/SDC  + DNase I | decrease  DNase I *vs.* native | *p* value | decrease  DNase I *vs.* SDS/SDC | *p* value | decrease  SDS/SDC *vs.* native | *p* value |
|  |  |  |  | |  |  |  |  |  |  |  |
| **UV-Vis** | tissue lysate | n.d. | n.d. | | n.d. | - | - | - | - | - | - |
|  | solid-phase | 1692 ± 191 | 1246 ± 219 | | 665 ± 87 | 60.7% | 0.004 | 46.7% | n.s. | 26.4% | n.s. |
|  | salting out | 4207 ± 498 | 1375 ± 343 | | 579 ± 109 | 86.2% | < 0.001 | 57.9% | n.s. | 67.3% | < 0.001 |
| **PicoGreen** | tissue lysate | 1223 ± 137.2 | 943.1 ± 143.3 | | 181.4 ± 36.6 | 85.2% | < 0.001 | 80.8% | < 0.001 | 22.9% | n.s. |
|  | solid-phase | 1324.9 ± 88.5 | 1191.7 ± 194.2 | | 269.8 ± 53.9 | 79.6% | < 0.001 | 77.4% | < 0.001 | 10.0% | n.s. |
|  | salting out | 1424.8 ± 89.4 | 898.8 ± 160.2 | | 138.2 ± 55.2 | 90.3% | < 0.001 | 84.6% | < 0.001 | 36.9% | 0.022 |
| **QPCR** | tissue lysate | n.d. | n.d. | | n.d. | - | - | - | - | - | - |
|  | solid-phase | 20.54 ± 0.44 | 22.10 ± 0.48 | | 23.92 ± 0.44 | +3.38 | < 0.001 | +1.82 | 0.013 | - | - |
|  | salting out | (17.97)* | (19.33 ± 1.20)* | | (19.21 ± 0.40)* | - | - | - | - | - | - |
| **DAPI** | image-based | 4.82 ± 0.55 | 2.43 ± 0.26 | | 0.43 ± 0.13 | 91.1% | < 0.001 | 82.4% | < 0.001 | 49.5% | 0.003 |
